# SPECIAL ISSUE The Promises and Pitfalls of “Clean Energy Transitions”: Introduction to the Special Issue

**DOI:** 10.3167/ares.2025.160101

**Published:** 2025-09

**Authors:** Dana E. Powell, Thomas A. De Pree

**Affiliations:** serves as associate professor in the Graduate Institute of Medical Humanities and Director of the Center for Humanistic Innovation and Social Engagement, College of Humanities and Social Sciences, Taipei Medical University (Taiwan).; research assistant professor in the School of Engineering and Affiliated Faculty in the Department of Anthropology at the University of New Mexico (USA).

## Clean Energy’s Contradictions

To avert the worst forms of climate catastrophe, the Intergovernmental Panel on Climate Change (IPCC) has called for immediate action toward achieving net-zero emissions of greenhouse gases that trap heat and warm the planet. In response, multiple forms of climate action and research, including emergent forms of climate modeling^1^, have manifested in response to calls for “clean energy transitions.” During such “transitions,” however, the existing social and ecological harms are often compounded rather than attenuated. What once seemed clean turns out to be entangled with violence of many kinds. It is now clear that so-called transition technologies are emerging not so much as alternatives with intrinsically liberatory outcomes but are often enacted by states and corporations to serve the status quo. The last decade has witnessed how the promise of renewable energy has become possible through coerced political dependency on the expansion of oil and gas extraction. Moreover, the moral discourse of “clean” energy technologies obscures the very dirty practices of industrialization and other seemingly post-industrial innovations. The rare earth and other minerals needed to generate batteries, photovoltaics, and other requisite components of the infrastructure can only be made possible by the rapid expansion of mining activities, which are closely associated with disparate health outcomes and labor rights abuses. Those who live downwind, downstream, and downgradient of the mines and other industrialized energy operations experience these contradictions firsthand. Contrary to the promises of a way out of modernity’s excesses, the pitfalls of clean energy raise critical questions for socioecological theory and environmental justice.

This collection of review articles^2^ draws attention to the *contradictory*^3^ as theory and method in humanistic and social science analyses of technology, through close engagements with controversies and conflicts that emerge at sites of transnational extractive capitalism and colonialism for the sake of “clean” energy. With the authors in this special issue, we contend that green ambitions must be held accountable for extending patterns of violence through global supply chains and local designs that deepen, rather than ameliorate, dispossession. They are often part and parcel of a broader logic of capitalist expansion (whether fossil-based or otherwise). What does “clean” come to mean in these contexts? What are the lived experiences of these technologies? And what and whose temporalities and spatial imaginaries of clean hold sway, shaping policies and cultural practices, across diverse communities? Promises of sustainability and safety, the standards and measures of clean, are contested through “the politics of baselining,” for example, which raises questions about what forms of contamination are deemed natural versus anthropogenic across categories of so-called stakeholders (De Pree 2020).

Our framing terms, “promises” and “pitfalls,” direct our attention toward an ethnography of promises and their contradictions. Mike Fortun’s work on promissory discourse calls for “an ethnography of ‘forward-looking statements’ and their performances in an international political economy” (2008: 11). How does hydropower, wind, solar, EVs, bioenergy, and even nuclear^4^ perform this discursive work of promising amidst the many contradictions that continue to unfold? We are thinking of promises in terms of speculative and effective discourse in the genesis and development of “technopolitical” energy regimes (Hecht 2012). The promissory discourse of “clean energy transitions” is extremely effective in capturing and foreclosing our collective futures, as this discourse foregrounds the seeming inevitability of a technological fix, rather than a more fundamental reconsideration of our relations and their continuance (see Whyte 2020). This promissory discourse (and its contradictions) can further be examined with the anthropological concept of ecological “prognosis,” which refers to new ways of modeling, planning, and forecasting the future of natural resources, as well as the messy interventions that make such plans become our shared reality (Kneas 2016: 83; Ferry and Limbert 2008; Mathews and Barnes 2016). We are using the word “pitfall” as opposed to “challenges,” “constraints,” “limitations,” “problems,” “failures,” and so on, because pitfalls are usually somewhat hidden. They catch us off guard. Pitfalls are often poorly camouflaged but ultimately serve as traps for those unsuspecting creatures that are not cautious, and perhaps a bit overly confident or distracted by other things. There’s something precautionary about promises and pitfalls, which makes us think about how “the precautionary principle” of taking preventative action in the face of scientific uncertainty (e.g., contamination’s casualities) serves as an understory within this collection, even if it is not explicitly mentioned throughout.

When viewed from the ground, the “forward-looking statements” offered by promises and pitfalls slip from their secure pedestals as discursive opposites, enfolding into one another. Claims of “clean” harbor the promissory *and* the precarious. The articles in this special issue move critical socioecological analytics away from theoretical reliance on binaries like clean or dirty, renewable or nonrenewable, infinite or finite. Rather, these projects stay with hybridity, complexity, transgression, and contradiction. In this collection, we find there is no such thing as absolutely “clean energy,” when viewed from the ground. The “promises and pitfalls” of what constitutes clean energy are not so much a check sheet of perks or perils, pros or cons, but deeply entangled, historically specific processes of accumulation, dispossession, and environmental injustice. Whereas “clean energy” as an idea once seemed to be a harbinger of brighter, healthier futures; its specific enactments all too often usher in—or collude with—“extractivisms,” or extractive ideologies and their toxic legacies. This special issue exposes these contradictions and complexities and ends up where Sartre (1955) did: There may be “no exit” from our existential quandaries, but there may yet be ways to (re)establish more ethical relations, by decentering overdetermined claims of “clean” to examine diverse localities and differently situated forms of transgression and transition.

We need to think across scales, re-humanizing (not only post-humanizing) what might at first glance seem to be a purely technoscientific matter. This collection of projects aligns STS with political ecology and anthropology to expose what are often the hidden technopolitics and embodiments of new energy economies. They also move to link seemingly disparate ecologies, taking a justice-centered approach to examine what lithium, nickel, uranium, hydro, EVs, and detoxification endeavors have to do with human rights and social justice. This collection of articles traces the conceptual and empirical contours of contradiction by following extractivism when and where it takes a “renewable” form—while opening this term to reveal the counter-posed imperatives of mitigation and adaptation strategies. By “extractivism” we refer not only to development projects of large-scale intensive mining and natural resource depletion and exhaustion but also to a broader ideology that is so pervasive it makes us all complicit to some degree or another (Jacka 2018; Jalbert et al. 2017; Powell 2017; Willow 2018). With global aspirations for developing renewable and nuclear energy capacities in response to planetary ecological crisis and the growth of independent states, there is a growing demand for “critical and strategic” minerals, which will require an expansion of extractive industries worldwide with serious implications for ecologies and their constitutive relations. Indeed, mining is predicted to increase by more than 500 percent to meet current low-carbon energy goals.^5^ So-called renewable energy technologies are sources of energy that are not depleted when they are used (e.g., solar panels and wind turbines) but require minerals that can be exhausted, even though they can be salvaged too. Lithium is required for the development of lithium-ion batteries that are essential for energy storage, and rare earth metals are necessary in the manufacturing of solar panels. Copper demand will increase with such growing forms of electrical generation. Geothermal and hydropower often demand lands, waters, heat, and steam in rural places where enclosures by states and industry have long undermined local autonomy and health. In other cases, such as bioenergy and emergent GHG-capture technologies, the expansion of land-intensive, industrialized modes of agro-forestry or livestock production is required, entrenching forms of social, ecological, and public health impacts in landscapes that have long accumulated the harms of late industrial capitalist extraction, racism, and colonialism, as many of the articles in this special issue explore. And yet, as the US administration pushes an aggressive return to fossil-gas resources (at the time of this writing), constructing nonviolent alternatives feels particularly imperiled. What is the role of critical environmental theory and engaged research, at this juncture? With the authors in this issue, we call for grounded engagement with this dilemma.

## Rethinking Transition

The “transitions” literature in the context of climate studies refers to low-carbon transitions and renewable energy transitions and transition management in public policy (Rotmans et al. 2001). Far from a simplistic, binary moral grammar of energy (i.e., “renewable” is good versus “nonrenewable” is bad), we hope to show and engage in an unapologetic examination of ways in which extractivism in its diverse, multivalent forms continues as a global, hegemonic norm. This extractive logic—indeed, it is nearly a cosmology of its own—is driven by the unbridled desire to accumulate and control, incarcerate and expel; it is part and parcel of a planetary politics in which authoritarianism meets the Anthropocene, attempting to re/organize and command natural resources, human resources, and territory for the expansion of empire.

In this volatile political context, these local stories of lithium, hydropower, electric vehicles, and solar projects may seem inconsequential or mundane. They are, however, the material manifestation of competing forms of developmentalism, expansion, and accumulation by dispossession *as well as* glimpses of other forms of resistance, resilience, and environmental world-making in an anticipatory politics of transformation (Kneas 2016), or “undone science” often taken up by social movements engaging with industrial forms of harm (Hess 2016). Rethinking transition, we use the prefix “trans-” (across, beyond, through) in the broadest sense to index the potentially limitless directions and extent to which one could go with the concept of “transitions” considering a climate emergency that calls for demolition, mitigation, and reclamation, though is often imaginatively bereft (see Powell 2025). In this special issue, we are considering how the trans- in social and environmental analytics that are trans-national, trans-capital, and trans-disciplinary help us recognize the transcendentalism of speculative visions, as well as promissory and anticipatory forms of discourse and practice.

This collection also engages with “just transitions” that bring about more than just technological change. Just transitions are only made possible by transgressing and changing values: not just shifting from one form of extractive technology or mineral resource to another (e.g., from coal to solar) but going against dominant forms of oppression and refusing the ontological designs of empire. In that sense, transition can become more than a linear move, or a continuation of the steady march of progress. Thinking of bell hooks’ emphasis on “the importance of intellectual work” in her book, *Teaching to Transgress*, how can critical climate studies of transitions recognize “the production of theory as a social practice that can be liberatory” (1994: 67)? Indigenous theories and practices are at the forefront of such radical cultural “transformation” beyond just a technological “transition” (IEN 2017). The IPCC defines and adopts “just transitions” as, “a set of principles, processes and practices that aim to ensure that no people, workers, places, sectors, countries or regions are left behind in the transition from a high-carbon to a low-carbon economy” (IPCC 2023). Discussions of transitions in climate science and policy can no longer ignore questions of justice. The concept of “just transitions” forces us to ask: *transitions for whom?* We need just transitions to remind us to teach to transgress. As we scale up our critical sensibilities of transition technologies and values, we need to be careful of the risks of co-optation and the ways cynical oil and gas think tanks take advantage of our critiques to pit us against one another, to stultify critical social movements and industrial transitions, and to promise more false solutions. We must watch out for this pitfall as well.

Although we do not claim authority to speak comprehensively or in totality about what counts as a “just transition,” and what that means and how it feels for communities affected by unjust transitions, this is the subject of many of the articles in this special issue. Thinking of “transitions” from an anthropological perspective conjures the binaries that frame discussions about continuity and change, contingency and disjuncture, mimesis and alterity, structure and agency. What does the notion of “transitions” (energy, environment, social, biological) reflect upon the longstanding engagement in history, anthropology, and the social sciences on continuity and change?

Given the track record of past performance by transnational mining companies, scholars and activists expect the results to include massive alterations of ecosystems, extreme social conflict, and environmental degradation (Jacka 2015; Kirsch 2014). The production of social and environmental health disparities, vulnerabilities, and injustices across rural Indigenous lands specifically—as sites of subterranean energy mineral wealth and water resources—and across overburdened communities generally, has been central to environmental justice scholarship as the cumulative impacts of multiple extractive industries become evident (Curley 2018, 2021, 2023; Emanuel 2024; Gutierrez et al. 2021; Lewis et al. 2017). Thus, “clean” energy transitions—as important as they are in attenuating the global impacts of anthropogenic climate change—continue to produce and ossify “sacrifice zones” as an inherent part of energy resource extraction programs. Even though environmental defenders working at the front lines of these urgencies articulated this impasse well before academics and continue to shape the critical discourse into which this set of articles intervenes, theoretical formulations remain an important climate action. As a set of dynamic and diverse inquiries into the everyday lives (human and otherwise) of “clean energy” systems, moving beyond just the technoscience, this set of articles interrogates the social, cultural, and political implications of “clean energy transitions.” This special issue of *Environment and Society* engages with diverse and dynamic responses to this provocation of “pitfalls and promises” with the goal of advancing decolonial and anti-capitalist, critical-theoretical and grounded-empirical analytics of renewable forms of extractivism.

## Renewing Collaborations

Ethnographic and qualitative approaches to transitions offer knowledges that are situated (Haraway 1988) and co-constituted (TallBear 2013). In curating our colleagues’ work and reflecting it back through our own shared and differently positioned lives and analytics, we bear the responsibility of editors and admit our roles as immodest witnesses. We are both shaped by our relations, and carry those traces of history, obligations, alignments, and experiences into the analytics we offer in this introduction and in the way we read and engage these articles. Transitions too, like our own perspectives, are situated.

As US citizens/white STS anthropologists, we have had the privilege to be in dialogue for many years with Diné collaborators in the Navajo Nation and the greater US Southwest, on issues of energy, nuclearism, environment, health, settler coloniality, and rurality. These shared relations and ongoing conversations shape our approach. Our commitments and responsibilities to collaborators, friends, and colleagues in extractive-intensive landscapes anchor our sense of place and sense of urgency in pointing to clean energy contradictions and potential transgressions. For Powell, this work began two decades ago in Window Rock, Navajo Nation, with uranium moratorium and radiation exposure activism targeting federal and tribal policies, expanding in 2006 into Eastern Agency chapters in response to the groundswell of regional concern over a proposed coal-fired power plant slated for construction near the San Juan River (New Mexico). The attending sovereignty and health concerns are widespread; co-authorship with Diné intellectuals on energy infrastructures has been one method, among others, to elevate these grounded analytics (Powell and Curley 2009; Powell and Becker 2022; Powell and Long 2023 [2010]; Powell and Tulley 2024). In other watersheds, Powell has worked collaboratively on extraction projects, with interdisciplinary academic and community-based partners through the Eastern North Carolina Environmental Justice Co-Lab, focusing on industrialized livestock-biogas and forest-based biomass infrastructures; this “Co-Lab” innovates new practices of research collaboration and knowledge co-production (Powell et al. 2024; Tulbure et al. 2024; Witter et al. 2025). Since 2021, Powell’s relocation to rural eastern Taiwan opened a space for translocal and collaborative methodologies to examine the impacts of “green growth” and surging environmental enthusiasm on Indigenous (esp. Bunun) mountains and rivers, within a geopolitical conflict zone.

For De Pree (2024), this work began over the course of the last decade, in collaboration with the Multicultural Alliance for a Safe Environment, which formed in response to the devastating impacts of uranium mining and the insufficient forms of expertise invested in monitoring and cleaning up abandoned mine lands across the “Grants Uranium District.” Over the last five years, De Pree has carried out collaborative ethnographic research among environmental health scientists and their community partners in the METALS Superfund Research Program at the University of New Mexico. These experiences shape and shade the way De Pree encountered the promise of nuclear energy as a “false solution” by tracing the dense entanglements of science, technology, and politics in cleaning up abandoned uranium mines and mill tailings that are leaching into Diné, Pueblo, *Nuevomexícano* (“Hispano/Indo-Hispano”), and white (“Anglo”) settler landscapes and waterways.^6^ De Pree teaches to transgress in anthropology and environmental science at Southwestern Indian Polytechnic Institute. Our respective and shared engagements and longstanding ethical commitments bind and guide our approach as guest editors.

We are also deeply informed—beyond what this introduction can convey—by the work of scholars in the environmental health and climate sciences, and activists in social movements for environmental justice and health equity, who show how historical problems of energy resource extraction are never simply technoscientific matters of development, but issues that center the urgencies of Indigenous self-governance. Andrew Curley (2023) reminds us of the radical contingency of “carbon sovereignty” in Diné environmental governance as coal takes on new meanings in a changing political economy; and Teresa Montoya (2017) examines extraction’s toxic legacies across Diné territories, from mine spills into local waterways to uranium mining’s radioactive legacies.^7^ Teracita Keyanna and colleagues (2025) recount emerging forms of Diné community-driven research in response to the combined threat of nuclear and mining disasters made manifest by “settler colonial determinants of health.” Our colleagues’ work, and our dialogues, shape how we as co-authors make sense of contradictions—conceptually, empirically, and politically.

## Toward a New Critical Transitions Literature

The articles in this issue invite critical examinations of “transition” technologies and values, and a more rigorous re-examination of the contexts, contours, and contradictions of lived experience within and against the infrastructures of extraction. Together, they draw our attention to the local histories and diverse embodiments of extractive economies and technologies, and the various modes of racial capitalism and coloniality that undergird and are reproduced by moves toward “clean energy” (Lennon 2020). As a set, the articles refresh anthropological and kindred theories of extractivism by taking nothing for granted; they begin from the generative tensions of contradictions and lay them bare. They complicate rather than settle, demonstrating the need for transgressive readings of seemingly apolitical scientific claims. Ultimately, they reveal how extractivism, as a practice and an ideology, continues to be remade, sustained, and even expanded, despite claims of its replacement. Their ethnographic and policy-oriented readings show collisions of “alternative value practices” (Whitington et al. this issue) as the ontological stability of “clean energy” erodes to reveal contingent, contested sites of struggle.

The articles in this special issue bring vitality to how new forms of natural resource extraction and waste mitigation in colonized, racialized, and capitalized geographies operate to undermine Indigenous sovereignty and racial justice. Many of the articles’ careful attention to questions of land and water relations, self-determination and matters of nation-building show a sensitivity to emerging critical theories in Indigenous political ecology (Carroll 2015; Carroll and Hunt n.d.; Middleton 2015). This emergent literature, based in land relations, avoids well-worn political ecological genealogies, instead striking out in novel conceptual directions, grounded in sustained and ethical relations with sites of extraction, pollution, *and* resilient innovation (see also Liboiron 2021). We align with this new literature in socioecological analytics that centers justice and Indigenous relationalities to understand historically- and geographically particular movements of colonialism, capitalism, and empire as these forces shape specific embodiments of harm and newly articulating modes of care.

Authors in this issue offer rich concepts for re-examining the lived experiences of resource depletion, mining, and mitigation efforts to refresh our analytics: these include “exhaustion” (Barandiaran, this issue), “body burdens” (Victor et al., this issue), and “ecologies of care” (Whitington et al., this issue). They expose the contradictions of the temporal promises of “clean energy,” revealing regressive strategies and “transitioning in reverse” (Philips and Dean, citing Günel, this issue), and the “entrenchments of climate colonialism” (Reed at al., this issue). The progressivist, liberal logics of renewables that appear to promise new futures for all, turn out to have hidden pitfalls across policy, practice, and populations where vulnerability is reproduced to meet national and global goals for GHG reduction. As a set, the articles illuminate how attending to local processes of collaboration and coloniality, as Libassi and authors say, reveals the sticking points and other impasses of climate mitigation. The articles span contemporary cases in Tanzania, Indonesia, urban and rural USA, Canada, and widely across Latin America, where “post-extractivism” discourses are being tested in practice. All too often, such discourses exacerbate “routine labors that produce cumulative dangers,” as Barandarian explains, through a deep examination of copper and lithium mining in Chile. Amy Zhang offers an ethnographic glimpse into waste-to-energy incineration in China and its exacerbation of existing inequalities and ignition of new forms of citizen science and environmental defense, widening the scope of articulations of empire and energy systems (see also Zhang 2024). Working across a range of democratic and authoritarian states, as well as within and across Native Nations and other polities, this set of articles does not take “the state” or “technology” as stable entities. Rather, they approach these as historically contingent formations through which environmental governance emerges as a key site of politics amidst the ongoing incursions of capitalism and ongoing processes of colonialism, reshaping human and wider ecologies.

Though pluralistic in voice, style, and location, these articles cohere in their challenge to the “banner of circularity” (to quote Victor et al.’s apt title), exposing exposure, and naming contradictions. These articles make a significant contribution to the interdisciplinary literature on environmental justice, by showing the diverse embodiments of harm and the problems of environmental governance across scales. All the articles agree in the urgency of centering social and environmental justice in conversations dominated by technoscientific fixations; for instance, carbon capture conversion and utilization infrastructures that transport distributive, procedural, and recognition forms of injustice (Tiwari et al. this issue). Victor et al. call these “technologies of unknowing,” as they operate to hide the true costs of waste-to-energy projects, a kind of concealment of the original contradiction; that is, they conceal the fact that the “clean” is in fact profoundly dirty, in this case). Apart from solely advancing unending critique, several of the articles move us collectively toward tracking and facilitating emerging innovations in decolonial social practice and policy, that emerge from grounded relations in specific places: as Reed et al. argue (this issue), “energy transitions must center decolonization as analytical framework; understand and act on the root causes driving the climate crisis; uplift Indigenous-led solutions and knowledge systems; and create space for Indigenous visions for Just Transitions.”

This issue builds upon longstanding ethnographic work and political engagement by environmental anthropologists, human geographers, and others in related fields, that laid the foundation for critical energy studies. As early as 1989, Laura Nader articulated the “barriers to thinking new about energy” among NASA scientists reluctant to engage with solar technology. Nader identified the hegemonic “groupthink” that stymied innovation and limited scientific imaginations. Since Nader’s foundational feminist critique, anthropologists have steadily argued for the urgency of anthropological intervention into energy issues. Ethnographic engagements grew and by the twenty-first century, critical energy studies was solidly secured, with new work on electrification (Gupta 2015; Winther 2010; Winther and Wilhite 2015), oil (Sawyer 2004), mining (Powell 2018), hydropower (Folch 2019; Hite 2022; Johnston 2017, 2018; Johnston and Fiske 2013), solar (Cross and Murray 2018, Lennon 2017, 2020, 2025; Yang 2015), agrofuels (McMichael 2009), and wind (Boyer 2019; Howe 2019) among many more anthropologists and geographers engaged in examining “cultures of energy” in ways attendant to history, power, culture, and meaning (see Strauss et al. 2013) and locally-grounded understandings of the “geosociality of energy transitions” (Whitington and Oguz 2024). The articles in this collection grow from these foundations and hone our attention to the contradictions of “clean” energy, possibilities for reading and teaching “transition” as transgression, and emergent sites of extractivist practice and assertions of relationality and revitalization of liberatory values.

These articles mark a turn in the field, from energy systems and politics of energy to a more specific, focused examination on renewability’s discursive claims, affective power, and lived, embodied experiences. As such, they also resonate with an emerging suite of projects in critical transition studies that attend to the intellectual and political value of ethnographic approaches to the energy crisis. As Banks et al. detail in this issue, low-carbon projects remain sites of socioecological injustice and despite a “growing archive of influential social science research on the energy transition,” there remains an urgent need for both policy and cultural analysis of the textured, lived dimensions of transition projects. Forthcoming edited volumes in critical transition studies (Willow and Abiral, 2025) and a special issue on extractivisms (Winchell and Howe, 2024). We are aware of numerous projects from eastern North Carolina to the US Southwest, and from Taiwan to Ecuador that are attending to clean energy’s contradictions and seeking new ways of understanding energy dilemmas, through anthropological theory and “extractivism’s limits” (Jobson et al., 2024). We are energized and honored to curate this special issue of *Environment & Society* and engage in these social movements.

## Notes:

1. See the anti-colonial and feminist experimental climate modeling of Steele et al. 2025.
2. A broad and vibrant community of practice informs our introduction. The opportunity to co-curate this special issue of *Environment and Society* emerged after Jerry Jacka and De Pree co-organized a panel on “clean energy transitions” (in which Powell presented a paper co-authored with Rebecca Witter) at the annual meetings of the American Anthropological Association (AAA) in Toronto in 2023. Authors DePree and Powell first met at the AAA meetings in Denver in 2015. Our collaborative engagements continued in Santa Fe at the Society for Applied Anthropology meetings in 2017 and we presented on environmental justice (EJ) and extractivism panels together at the Society for Social Studies of Science (4S) in Mexico City 2022 and 4S Honolulu 2023, with Kim Fortun’s EcoGovLab and Global EiJ Record project; these communities of practice inform our analysis. Powell is especially grateful to colleagues in the ENC EJ Co-Lab (Rebecca Witter, Donna Chavis, Jeff Currie, Sherri White-Williamson, Mac Legerton, and Danielle Melvin Koonce) for the collaborative, community-aligned research on bioenergy extractivism reshaping the rural Coastal Plains, which anchors her understanding of the “renewable ruse” of industrialized biomass and biogas (Powell et al. 2024; Witter et al. 2025). De Pree acknowledges the key contributions and leadership of his colleagues, Teracita Keyanna (Diné) and Kirena Tsosie (Diné), in a collaborative presentation at 4S 2023.
3. We use “contradiction” in the general sense of the term but acknowledge its specific use in critical geography (see David Harvey 2014).
4. Prospecting on the “key role” of nuclear energy in achieving global net-zero greenhouse gas emissions, the US Department of State (2023) recently published the Declaration to Triple Nuclear Energy (https://www.state.gov/declaration-to-triple-nuclear-energy). In comparison, even as Taiwan moves toward denuclearization and “green growth” in geothermal, wind, solar, and hydro sectors, some analysts argue that the island still “cannot shake its nuclear ghosts” (https://foreignpolicy.com/2024/03/14/taiwan-nuclear-energy-weapons-policy-history/).
5. https://www.un.org/en/climatechange/raising-ambition/renewable-energy-transition, accessed December 16, 2023; https://pubdocs.worldbank.org/en/961711588875536384/Minerals-for-Climate-Action-The-Mineral-Intensity-of-the-Clean-Energy-Transition.pdf, accessed December 16, 2023.
6. On “just transitions” and “false solutions,” and connecting theory and practice on these topics, I am inspired by the work of Pueblo Action Alliance and No False Solutions Coalition: https://www.puebloactionalliance.org/no-false-solutions; https://www.nofalsesolutions.com (accessed July 3, 2025).
7. Our earlier conversations with these collaborators, on conference panels and in other settings, inform the analysis here. Powell also recognizes an NSF Workshop Award on Toxicity and Transition in the Navajo Nation, co-led with Curley and Montoya from 2020 to 2021, which informs some of her thinking on Diné transition theory (though the findings from that project are not specifically discussed in this article).

## References

[R1] Boyer Dominic. 2019. Energopolitics: Wind and Power in the Anthropocene. Durham, NC: Duke University Press.

[R2] Carroll Clint. 2015. Roots of our Renewal: Ethnobotany and Cherokee Environmental Governance. Minneapolis: University of Minnesota Press.

[R3] Carroll Clint and Hunt/Tłaliłila’ogwa Sarah, eds. Forthcoming. Indigenous Political Ecology. Minneapolis: University of Minnesota Press.

[R4] Cross Jamie, and Murray Declan. 2018. “The Afterlives of Solar Power: Waste and Repair Off the Grid in Kenya.” Energy Research & Social Science 44: 100–109.

[R5] Curley Andrew. 2018. “A Failed Green Future: Navajo Green Jobs and Energy ‘Transition’ in the Navajo Nation.” Geoforum 88: 57–65.

[R6] Curley Andrew. 2021. “Infrastructures as Colonial Beachheads: The Central Arizona Project and the Taking of Navajo Resources.” Environment and Planning D: Society and Space 39 (3): 387–404.

[R7] Curley Andrew. 2023. Carbon Sovereignty. Tucson: University of Arizona Press.

[R8] De Pree Thomas. 2020. “The Politics of Baselining in the Grants Uranium Mining District of Northwestern New Mexico.” Journal of Environmental Management 268 (August): 1–8. 10.1016/j.jenvman.2020.110601 32510424

[R9] De Pree Thomas. 2024. “Origin Stories of the ‘Grants Uranium District’ in Northwestern New Mexico: Archives, Memoirs, and Exploratory Boreholes in the Production of Geological Regions.” Engaging Science, Technology, and Society 10 (3): 37–64. 40894256 10.17351/ests2023.2323 PMC12392682

[R10] Emanual Ryan. 2024. On the Swamp: Fighting for Indigenous Environmental Justice. Chapel Hill: University of North Carolina Press.

[R11] Ferry Elizabeth Emma, and Limbert Mandana E.. 2008. Timely Assets: The Politics of Resources and Their Temporalities. Santa Fe, NM: School for Advanced Research Press.

[R12] Folch Christine. 2019. Hydropolitics: The Itaipu Dam, Sovereignty, and the Engineering of Modern South America. Princeton, NJ: Princeton University Press.

[R13] Fortun Mike. 2008. Promising Genomics. Oakland: University of California Press.

[R14] Gutierrez Grant M., Powell Dana E., and Pendergrast TL. 2021. “The Double Force of Vulnerability: Ethnography and Environmental Justice.” Environment and Society: Advances in Research 12 (1): 66–86.

[R15] Gupta Akhil. 2015. “An Anthropology of Electricity from the Global South.” Cultural Anthropology 30 (4): 555–568.

[R16] Haraway Donna. 1988. “Situated Knowledges: The Science Question in Feminism and the Privilege of Partial Perspective.” Feminist Studies 14 (3): 575–599.

[R17] Harvey David. 2014. Seventeen Contradictions and the End of Capitalism. Oxford: Oxford University Press.

[R18] Hecht Gabrielle. 2012. Being Nuclear: Africans and the Global Uranium Trade. Cambridge, MA: MIT Press.

[R19] Hess David. 2016. Undone Science: Social Movements, Mobilized Publics, and Industrial Transitions. Cambridge, MA: MIT Press.

[R20] Hite Emily. 2022. “The Many-Headed Hydra: Assessing the Indigenous-Hydropower Cycle in Costa Rica.” Journal of Political Ecology 29 (1): 656–671.

[R21] hooks bell. 1994. Teaching to Transgress: Education as the Practice of Freedom. New York: Routledge.

[R22] Howe Cymene. 2019. Ecologics: Wind and Power in the Anthropocene. Durham, NC: Duke University Press.

[R23] Indigenous Environmental Network (IEN). 2017. “Indigenous Principles of Just Transition.” https://www.ienearth.org/wp-content/uploads/2017/10/IENJustTransitionPrinciples.pdf.

[R24] Intergovernmental Panel on Climate Change (IPCC). 2023. AR6 Synthesis Report. https://www.ipcc.ch/report/ar6/syr/.

[R25] Jacka Jerry K. 2015. Alchemy in the Rain Forest. Durham, NC: Duke University Press.

[R26] Jacka Jerry K. 2018. “The Anthropology of Mining: The Social and Environmental Impacts of Resource Extraction in the Mineral Age.” Annual Review of Anthropology 47 (1): 61–77. 10.1146/annurev-anthro-102317-050156.

[R27] Jalbert Kirk, Anna Willow, David Casagrande, Stephanie Paladino, and Jeanne Simonelli, eds. 2017. ExtrACTION: Impacts, Responses, and Alternative Futures. Walnut Creek, CA: Left Coast Press/ Routledge.

[R28] Jobson Ryan Cecil, Macarena Gómez-Barris Cymene Howe, and Winchell Mareike. 2024. “Extractivism’s Limits: A Conversation.” Journal of Latin American and Caribbean Anthropology 29 (3): 255–260.

[R29] Johnston Barbara Rose. 2017. ”Action-Research and Environmental Justice: Lessons from Guatemala’s Chixoy Dam.” In Routledge Companion to the Environmental Humanities, ed. Ursula Heiseand Christensen Jon,, 174–184. New York: Routledge.

[R30] Johnston Barbara Rose. 2018. “Large-Scale Dam Development and Counter Movements: Water Justice Struggles Among Guatemala’s Chixoy Dam.” In Water Justice, ed. Boelens Rutgerd, Perreault Tom, and Vos Jeoren, 169–186. Cambridge: Cambridge University Press.

[R31] Johnston Barbara Rose, and Fiske Shirley J.. 2013. “The Precarious State of the Hydrosphere: Why Bio-cultural Health Matters.” Wiley Interdisciplinary Reviews: Water. doi: 10.1002/wat2.1003.

[R32] Keyanna Teracita, Thomas De Pree Chris Shuey, Tsosie Kirena, Quetawki Mallery, and Jim Cheryl. 2025. “What Is “Restorative Justice” After the Church Rock Uranium Mill Spill?” Journal of Disaster Studies. PMC13052356 41947904

[R33] Kirsch Stuart. 2014. Mining Capitalism: The Relationship between Corporations and Their Critics. Oakland: University of California Press.

[R34] Kneas David. 2016. “Subsoil Abundance and Surface Absence: A Junior Mining Company and Its Performance of Prognosis in Northwestern Ecuador.” Journal of the Royal Anthropological Institute 22 (S1): 67–86. 10.1111/1467-9655.12394.

[R35] Lennon Myles. 2017. “Decolonizing Energy: Black Lives Matter and Technoscientific Expertise Amid Solar Transitions.” Energy Research & Social Science 30: 18–27.

[R36] Lennon Myles. 2020. “Postcarbon Amnesia: Toward a Recognition of Racial Grief in Renewable Energy Futures.” Science, Technology, and Human Values 45 (5): 934–962.

[R37] Lennon Myles. 2025. Subjects of the Sun: Solar Energy in the Shadows of Racial Capitalism. Durham, NC: Duke University Press.

[R38] Lewis Johnnye, Hoover Joseph, and MacKenzie Debra. 2017. “Mining and Environmental Health Disparities in Native American Communities.” Current Environmental Health Report 4 (April): 130–141. 10.1007/s40572-017-0140-5. PMC5429369 28447316

[R39] Mathews Andrew S., and Barnes Jessica. 2016. “Prognosis: Visions of Environmental Futures.” Journal of the Royal Anthropological Institute 22 (S1): 9–26. 10.1111/1467-9655.12391.

[R40] McMichael Philip. 2009. “The Agrofuels Project at Large.” Critical Sociology 35 (6): 825–839.

[R41] Middleton Beth Rose. 2015. “Jahát Jatítotòdom: Toward an Indigenous Political Ecology.” In The International Handbook of Political Ecology, ed. Bryant Raymond K., 561–576. Northampton: Edward Elgar Publishing.

[R42] Montoya Teresa. 2017. “Yellow Water: Rupture and Return One Year after the Gold King Mine Spill.” Anthropology Now 9 (3): 91–115.

[R43] Nader Laura. 1989. “Barriers to Thinking New About Energy.” Physics Today 34 (2): 9–104.

[R44] Powell Dana E. 2017. “Toward Transition?: Challenging Extractivism and the Politics of the Inevitable in the Navajo Nation.” In ExtrACTION: Impacts, Responses, and Alternative Futures, ed. Jalbert Kirk, Willow Anna, Casagrande David, Paladino Stephanie, and Simonelli Jeanne, 211–226. Walnut Creek, CA: Left Coast Press.

[R45] Powell Dana E. 2018. Landscapes of Power: Politics of Energy in the Navajo Nation. Durham, NC: Duke University Press.

[R46] Powell Dana E. 2024. “Life Beyond Ruin: Diné Presence in the Anthropocene.” Native American Indigenous Studies (NAIS) 11 (1): 71–113.

[R47] Powell Dana E. 2025. “The Transit of Transition: Three Conditions.” In Futures Post-Carbon, ed. Anna Willow and Bürge Abiral, Chapter 11. New York: Routledge.

[R48] Powell Dana E. and Tulley Earl. 2024. “Climate Change in a Time of COVID-19: Living and Transforming the Syndemic in the Navajo Nation.” In Stranger than Paradise: Reconceptualizing Globalization, ed. Hsien-hao Liao Sebastian. Taipei: National Taiwan University Institute for Advanced Studies in the Humanities and Social Sciences.

[R49] Powell Dana E. and Long Dáilan J.. 2023 [2010]. “Landscapes of Power: Renewable Energy Activism in Diné Bikeyah.” In Sustaining Natures: an Environmental Anthropology Reader, ed. Osterhoudt Sarah R. and Sivaramakrishnan K. Seattle: University of Washington Press.

[R50] Powell Dana E. and Becker Bidtah. 2022. “Situating Energy Justice: Storytelling Risk and Resilience in the Navajo Nation.” In Energy Democracies for Sustainable Futures, ed. Nadesan Majia, Pasqualetti Martin, and Keahey Jennifer, 215–224. Amsterdam: Elsevier.

[R51] Powell Dana E. and Curley Andrew. 2009. “*K’e, Hozhó*, and Non-Governmental Politics on the Navajo Nation: Ontologies of Difference Manifest in Environmental Activism.” World Anthropologies Network 4: 109–138.

[R52] Powell Dana E., Currie Jefferson, Koonce Danielle M., Legerton Mac, and Witter Rebecca. 2024. “Renewable Ruse: Bioenergy Development in North Carolina’s Coastal Plains.” Engaging Science, Technology, and Society 10 (1–2): 32–68.

[R53] Rotmans J, Kemp R and van Asselt M (2001). “More Evolution than Revolution: Transition Management in Public Policy.” Foresight 3 (1): 15–31. 10.1108/14636680110803003.

[R54] Sartre Jean-Paul. 1944 (1955). No Exit. New York: Vintage.

[R55] Sawyer Suzana. 2004. Crude Chronicles. Durham, NC: Duke University Press.

[R56] Steele Harlin/Hayley, McCullough Sarah Rebolloso, De La Torre Alexis, Caballero Avidane Ceana Aleman, Zhong Clara, Wei James, Moco Kalon Cecelia, Kotha Miguel, Meghana, Nguyen Thy, and Bowen Tess, . “Interactive Exhibit: Towards feminist, anti-racist climate modeling” in the “Making and Doing” Central Exhibition at the 50th Annual Meeting of the Society for Social Studies of Science (4S): Reverberations, Seattle, WA, 4 Sept 2025.

[R57] Strauss Sarah, Stephanie Rupp, and Love Thomas, eds. 2013. “Introduction.” Cultures of Energy: Power, Practices, Technologies. Walnut Creek, CA: Left Coast Press.

[R58] TallBear Kim. 2013. Native American DNA: Tribal Belonging and the False Promise of Genetic Science. Minneapolis: University of Minnesota Press.

[R59] Tulbure Mirela G. Júlio Caineta, Cox Brooke, Stehman Stephen V., Ercumen Ayse, Witter Rebecca, Emanuel Ryan, Powell Dana E., Burdette Kemp, White-Williamson Sherri, and Tuberty Shea, . 2024. “Earth Observation Data to Support Environmental Justice: Linking Non-permitted Poultry Operations to Social Vulnerability Indices.” GeoHealth 8 (12): 1–17. 10.1029/2024GH001179 PMC11652945 39697399

[R60] US Department of State. 2023. “Declaration to Triple Nuclear Energy.” Bureau of International Security and Nonproliferation. https://www.state.gov/declaration-to-triple-nuclear-energy/.

[R61] Whitington Jerome, and Oguz Zeynep. 2024. “Mining the Sun: Coal-to-Solar Transitions and Energetic Place-Making in Appalachia.” Critique of Anthropology 44 (3): 276–293. 10.1177/0308275X241269614.

[R62] Whyte Kyle. 2020. “Collective Continuance.” In 50 Concepts for a Critical Phenomenology, ed. Weiss Gail, Murphy Ann V., and Salamon Gayle, 53–60. Evanston, IL: Northwestern University Press.

[R63] Willow Anna J. 2018. Understanding ExtrACTIVISM: Culture and Power in Natural Resource Disputes. New York: Routledge.

[R64] Anna Willow, and Abiral Bürge, eds. 2025. Post-Carbon Futures. New York: Routledge.

[R65] Winchell Mareike, and Howe Cymene. 2024. “Unsettling Extractivism: Indigeneity, Race, and Disruptive Emplacements.” Journal of Latin American and Caribbean Anthropology 29 (3): 201–207.

[R66] Winther Tanja. 2010. The Impact of Electricity. New York: Berghahn Books.

[R67] Winther Tanja, and Wilhite Harold. 2015. “Tentacles of Modernity: Why Electricity Needs Anthropology.” Cultural Anthropology 30 (4): 569–577.

[R68] Witter Rebecca, Woods Courtney, Legerton Mac, Emanuel Ryan, Sherri White-Williamson Donna Chavis, Currie Jefferson II, Koonce Danielle M., Powell Dana E., Grooms Seth, Tuberty Shea, Christine Ogilvie Hendren Maggie Sugg. 2025. “Advancing an Environmental Justice-Centered Approach to Understanding Cumulative Impacts: Community-Engaged Analysis of Industrialized Biogas Development from Rural Eastern North Carolina.” Environmental Justice. 10.1089/env.2025.0009.

[R69] Yang Hung-Jen. 2015. “Reassembling Solar Farms, Reassembling the Social: A Case Study of Ping-Tung County in Southern Taiwan.” East Asian Science, Technology and Society: An International Journal 9 (4): 359–379.

[R70] Zhang Amy. 2024. Circular Ecologies: Environmentalism and Waste Politics in Urban China. Redwood City, CA: Stanford University Press.

